# Care Plan Templates in Adult Community Mental Health Teams in England and Wales: An Evaluation

**DOI:** 10.3390/nursrep14010026

**Published:** 2024-02-01

**Authors:** Donna Kemp, Michael Doyle, Mary Turner, Steve Hemingway

**Affiliations:** School of Human and Health Sciences, Queensgate Campus, University of Huddersfield, Huddersfield HD1 3DH, UK; m.doyle2@hud.ac.uk (M.D.); m.turner@hud.ac.uk (M.T.); s.j.hemingway@hud.ac.uk (S.H.)

**Keywords:** community mental health services, evidence-based practice, nursing process, patient care planning, universal design, user-centred design

## Abstract

Adults accessing community mental health services are required to have a care plan, developed in collaboration with the person accessing the service. The variation in care plan templates in use in England and Wales, and their impact on care planning, is unknown. This study evaluates the community mental health care plan templates in use across England and Wales. Data were obtained from a Freedom of Information request to 50 NHS Mental Health Trusts. An evaluation tool was designed and used to extract data. Data were rated red, amber, or green against clinical and design standards. Forty-seven care plan templates were obtained. The clinical aspect of the care plan template had 60% adherence to the national standards, and the design aspects had 87% adherence. A ‘high/low’ typology is proposed against the design/clinical standards. The study identifies priority areas for improvement in the care plan templates as space to record the actions that service users and carers will take to contribute to their care plan, space to record the name and contact details for their care coordinator or lead professional, plus others involved in the person’s care. This study was not registered.

## 1. Introduction

Within UK and health services worldwide, processes followed by health care professionals determine the health needs and treatment options for people accessing care. This involves assessing the person in order to understand their strengths and needs, working with the person and their carer to agree what will be done (care planning) and writing it down (a care plan), implementing the care plan, and then evaluating progress against the care plan [1,2]. This process is followed internationally [3,4,5]. For this article, the care plan is the subject of interest. The recording of a care plan is a professional requirement for nurses and allied health professionals [6,7]. A care plan is a written document that details the plan of care, support and treatment for the individual. Wrycraft [8] defines it as “a description of the interventions and actions that will occur in the person’s care; an outline of the desired outcome(s) against which progress is measured; a record of the agreement of the service user and mental health nurse, along with the date for review of each care intervention”.

Within the UK, mental health services are currently transitioning from a model of Care Programme Approach (CPA) to the Community Mental Health Framework (CMHF) [9]. The framework seeks to retain the ‘sound theoretical principles based on good care coordination and high-quality care planning’ of CPA embedding this within the CMHF [9]. The CMHF is based upon five broad principles: a shift in prominence from care coordination to compassionate, intervention-based care; a named key worker for all service users, replacing the care coordinator/lead professional role of CPA; high quality, co-produced holistic, personalised care and support planning for people with severe mental health problems living in the community; better support for and involvement of carers; and a more accessible, responsible, and flexible system [9]. The requirement of a care plan for service users remains prominent within the CMHF, with an emphasis on service user and carer involvement, the care plan being concise and accessible in a digital format.

The national guidance for community mental health in England does not mandate the design of the care plan, but rather that it simply exists. The CPA, CMHF and good practice guidelines refer to what should be included in the care plan, for example, service user preference, goals, interventions, and review date; however, they do not make explicit what the design of the care plan should be [9,10,11,12]. The result of this is that in England, there is a plethora of different care plan templates in use across National Health Service (NHS) Trusts, and similarities and differences are unknown. In contrast, Wales does mandate a care plan template, the Care and Treatment Plan [13,14]. A plethora of care plans may contribute to variation in service user and carer experience across different NHS Trusts, reflecting local care planning practices. The 2022 National Community Mental Health Survey, undertaken annually across NHS Community Mental Health Trusts, reveals that only 55% of people reported that they were definitely involved as much as they wanted to be in planning their care [15]. The lack of involvement of service users involved in their care is echoed across the world [16,17,18]; this is despite efforts to drive collaborative working and shared decision making in care planning [19,20]. The care plan template, in essence, a tool provided to clinicians with which to enable collaboration with service users, resides outside the evidence base. The impact of care plan design on the service user experience remains unknown.

The aim of the study was to establish a typology of care plan templates currently in clinical use across secondary NHS Adult Community Mental Health Teams in England and Wales. The three objectives to meet the overall aim of the study were to:

1. Develop an evaluation tool for evaluating care plans based on best practice evidence for design in clinical practice;
2. Collect samples of care plan templates being used in NHS community mental health teams in England and Wales;
3. Evaluate samples of care plan templates being used in NHS community mental health teams in England and Wales.

## 2. Materials and Methods

There are 50 NHS trusts in England and Wales who provide community mental health services to working aged adults [21]. Trust details were accessed via the Care Quality Commission (CQC), an independent regulator of health and social care in England. All NHS trusts were included in the sample.

Initially, each of the NHS Trusts’ research and development departments were approached by email, requesting a blank copy of the care plan template used in adult community mental health services. The response rate was under 25%. A further request was made via the Great Britain Freedom of Information Act 2000 again requesting the blank care plan template and, additionally, the name of the electronic health record (EHR) used by the Trust. This additional request was made as early analysis of the care plan template revealed variation in format of the care plan template. Some Trusts sent screenshots of the EHR; others sent the care plan template as it would be printed out. Understanding the range of EHR’s in use may help explain the variation in care plan templates provided, as not all have the capability of printing out a blank template.

A 7 section, 34-question evaluation tool was developed to evaluate each care plan template against specific standards associated with both the clinical care plan and interaction design.

The clinical care plan standards were derived from the National Institute for Health and Care Excellence quality standards [10] and NHS England policy guidelines [9,12] and are detailed within the data collection tool—see Table 1.

Nielson’s ten usability heuristics [23] informed the development of the tool (see Table S1). Cited as foundational in contemporary interaction design [24], Nielson’s heuristics are broad and applicable to any type of screen-based user interface, including character-based and graphical user interfaces [25]. Preece, Hill [26], in their heuristic evaluation of hospital observation charts, noted that there were no published usability heuristics available for observation charts or for any paper-based medical chart. Whilst observation charts do differ from care plans, there are also some similarities. The similarities are that both are designed locally; there are guidelines for what should be covered but no national template. Both are used in a paper format; the observation chart is filled in by hand, the care plan is completed either by hand and then input into the electronic health record (EHR) or directly completed in the EHR. Both are used by care coordinators/key workers and require being completed accurately; the observation chart is data heavy whereas the care plan is mainly narrative. Despite these differences, both are care records with professionals accountable for their accurate completion [6,7]. The eight headings utilised by Preece, Hill [26] were drawn upon to form the structure of the evaluation tool.

The tool was piloted on the first 12 care plan templates obtained. Some Trusts provided the care plan template as the clinician would use it within the electronic health record; this is referred to as an ‘input care plan template’. The input care plan templates had some design elements that could not be evaluated, specifically the page layout, information layout (part), use of fonts and use of colour. Other Trusts provided the care plan template as a ‘printed out’ care plan that the service user would receive, which is referred to as an ‘output care plan template’. Some Trusts provided both versions of the care plan. In these instances, the ‘output care plan template’ was used as all elements of the evaluation tool could be applied. Subsequently, the tool was adapted to evaluate the two different formats of care plan templates provided.

Each evaluation tool question was phrased to elicit a positive response; this was to assist in analysis and served to highlight positive practice. A notes column for each question allowed for commentary as necessary. The tool can be seen in the Section 3.

The data were analysed after the evaluation tool was scored by the researcher and checked by the research team members. Each single response was rated as meeting the standard or not meeting the standard. The results were then added within each of the seven sections and given an overall percentage score to achieve a compliance percentage. Compliance percentages were then rated as red where the results are 0% to 49%, amber where the results are 50% to 79% and green when the results are over 80%. Red, amber, and green (RAG) ratings were used as priority indicators for users of the evaluation tool. The Healthcare Quality Improvement Partnership [27] recommends the use of the ‘traffic light’ system as a way of identifying priority areas, stipulating a key as to the cut-off compliance rates. Caution is advised in allocating green to anything other than 100% [27]; however, the results show that only 3 areas scored 100%, indicating that the ‘green’ spread is broader than 100% for this study, hence the 80% threshold.

The SQUIRE [28] reporting guidelines for quality improvement in health care were used. The University’s School of Human and Health Research Ethics Panel reviewed the evaluation application and was approved (SREP-2019-097).

## 3. Results

Of the 47 NHS Trusts providing care plan temples, 44 of them provided the name of the electronic health record that they use. Six different systems were reported: Rio, SystmOne, CareNotes, PARIS, Lorenzo and Care Director. One NHS Trust used paper records. The mandated care plan used in Wales was included.

A total of 12 care plans were initially obtained following the informal approach to the 50 NHS Trusts. A further 35 care plan templates were provided after a freedom of information (FOI) request was made to the Trusts. The overall response was 94%.

Of the 47 care plans templates obtained, 2 of them were omitted, 1 as it was for older adults and the other as it was a care plan template sent in error. Therefore, 45 care plan templates were included within the analysis and 14 were input care plan templates, i.e., they showed the user interface for inputting the care plan to an electronic health record. The remaining 31 templates were output care plan templates; they were the versions to be presented to the service user. Results are presented as percentages of the care plans that adhered to the standard; see Table 2 for summary results.

### 3.1. Care Plan Clinical Content

All care plans were evaluated against the standards associated with care plan content (*n* = 45); 11 questions were applied—see Table 3.

Two thirds of care plan templates (66%) did not include a space to record the actions that the service user and carer would take as part of their care plan. This contrasts with 95% of care plan templates having space for the interventions offered by services to be recorded. Eighty two percent of care plan templates invited the recording of service user goals.

Though 57% of care plan templates recorded the name of the care coordinator/key worker and their contact details, only 45% requested the names and contact details of others involved in the person’s care. Fewer than half of the care plan templates had a place to record who the person’s carers or informal supporters were.

### 3.2. Page Layout

There were five questions pertaining to page layout—see Table 4. It was only possible to apply the tool where the NHS Trust had provided ‘output’ care plans (*n* = 31), i.e., those that would be printed out, rather than ‘input’ care plans, which follow the template as shown on the Electronic Health Record.

The care plans were presented well, with horizontal writing; superfluous information was omitted, and the layout was balanced, with white borders. However, 51% of the care plans were presented in portrait rather than the desired landscape orientation. Logos were not always discrete; indeed, many logos were missing rather than being non-discrete.

### 3.3. Information Layout

It was only possible to determine the information layout on the ‘output’ care plans (*n* = 31)—see Table 5. This section comprised six questions and each scored highly, with consistent adherence to the standard.

Sufficient writing space scored 100% as all care plan templates were intended for completion electronically and text boxes expand to accommodate the volume of text.

### 3.4. Language and Labelling

All care plans were evaluated for language and labelling (*n* = 45)—see Table 6. This section comprised five questions with an overall 77% adherence to the standards.

Personal pronouns are the optimum position to take in addressing the service user. Fifty five percent of the care plans adhered to this standard. The remaining 45% of care plans used alternative pronouns, for example: ‘patient’ (CP34) and ‘client’ (CP21). There were examples of where the use of pronouns changed throughout the care plan template, for example, alternating between ‘the person’ and ‘I’ (CP1).

The use of jargon, abbreviations and technical language was evident within 21% of the care plans; examples include ‘hospitalisation’ (CP14), ‘clinical rationale for planned intervention’ (CP13), ‘clinical impression and risk formulation’ (CP19), MHA, DoLS, MCA (CP1, 4), ‘1st, 2nd, 3rd line interventions, MBT, OCD, CBT, EMDR’ (CP15), ‘IHTT ALPS’ (CP33), and ‘THRIVE needs-based grouping’ (CP42). The Plain English Campaign advocates for the use of plain English in written communication [29].

Grammatical errors were found in 24% of the care plan templates. Examples included inconsistent use of hyphens, colons, and semicolons (CP6), ‘What would you like to de doing in the future?’ (CP8), ‘Have your carer been given a copy of Your Care Plan?’ (CP12), random initial capitalisation (CP20, 17,31,32,36), spelling error (CP31, 36), use of √ instead of 🗸 (CP31).

### 3.5. Cognitive and Memory Load

All care plans were evaluated for effective use of tick boxes (*n* = 45)—see Table 7. Preece, Hill [26] identify that writing within the electronic health record should not be required when responses could be provided via a tick box.

### 3.6. Use of Fonts

It was not possible to evaluate the use of fonts where the care plan template was within the Electronic Health Record. Reports produced from the EHR can be written to specify the font and point size, so it is not reasonable to evaluate based on the ‘input’ only (*n* = 14)—see Table 8.

Only 39% of care plan templates had the header point size the same as that of the rest of the care plan template. Headers should be a bigger point size than the main text for reader comfort, separating sections.

### 3.7. Use of Colour

The ‘output’ care plan templates were evaluated for contrast between the type and the background (paper) (*n* = 35)—see Table 9.

Most care plans were presented in black and white.

## 4. Discussion

The care plan templates were viewed as comprising two aspects: design and clinical. The results of the care plan template evaluation highlight that the care plan clinical content (in essence, the clinical aspect of the care plan) is the main area where improvements might be made. When the care plans are considered collectively, the clinical aspect was evaluated to have 60% adherence overall to the national standards compared with an overall 87% adherence to the design standards.

### 4.1. Clinical

The Community Mental Health Survey (CMHS) is commissioned by the Care Quality Commission annually to look at the experiences of people who use community mental health services across England and Wales. In 2022, the CMHS reported that 69% of service users had been told who their care coordinator or lead professional is; this number is down from 77% in 2014 [15]. The quality standard is that people are told the name of their care coordinator or lead professional and how to contact them [22]. In Queensland, Australia, care planning is a key performance indicator [19] and the Australian Commission of Safety and Quality in Health Care are explicit in stating the standards associated with collaboratively developing the care and recovery plan [30].

The results from the evaluation of care plan templates found that 43% of templates did not provide a place for the name and contact details of the care coordinator/lead professional to be recorded. This is important as it may provide some explanation to why 31% of people are reporting in the CMHS that they do not know who their care coordinator/lead professional is, or how to contact them. There are further considerations to be made; for example, if there was a place to record this information, it does not mean that a clinician would complete the information, unless it was made a mandatory field. Similarly, the evaluation of care plan templates found that 55% did not provide space for recording the names and contact details for others involved in the person’s care. Again, the national standard is that people do know this information [22].

Though service users and carers are found to want and value relevant information in their care plans [31,32], it remains a concern that people continue to report that they do not have access to their care plan [19,33]. Of the care plan templates evaluated, 48% did not provide a space to indicate if the service user had been offered a copy of their care plan. As already noted, this could be because this information is held in the EHR and suppressed from the care plan report (printed care plan), thus suggesting that this information is deemed as not relevant to the service user. Of this, it is not known as to who determines what is included in the printed care plan and what is omitted. Typically, an informatics team would be responsible for creating the report; however, it is perhaps worthy of exploration as to who contributes to this decision making process. Sharp, Rogers [24] recommend that interaction design, encompassing report writing and template design, should be undertaken as a multi-disciplinary pursuit, recognising that different stakeholders bring different requirements and ideas for solutions. In addition, different skill sets are required to ensure that the design is fit for purpose—clinical, technical, business, training and marketing [24]; thus, for the care plan template and the report, it would be prudent to include clinicians, service users and carers as well as the organisational development team and the informatics team in decision making. A pluralistic/heuristic and cognitive walk-through involving clinicians as well as developers and usability experts would serve to examine the process, attend to the detail, and identify and resolve issues ahead of release in practice [24,34,35].

The care plan templates were evaluated positively in terms of having space for recording the service users’ goals and what the service actions are. This is perhaps not surprising as it reflects what has been traditionally known as fundamental to a care plan, regardless of clinical speciality or setting [36]. A word of caution is advised by Barrett and Linsley [37], who recognise that goal setting and planning can serve to underline deficits, rather than strengths, thus working in opposition to recovery orientated care. All quality aspects of the care plan template were subject to evaluation, resulting in a holistic view of its overall strengths and areas to develop. A balanced and coherent care plan template, with a consistent voice, could be achieved should Trusts respond to the evaluation of their care plan template.

National quality standards in the UK place emphasis on shared decision making, where service users and carers are equal partners in planning care and the co-production of care plans [22]. Despite this, neither are consistently facilitated and captured within the sample of care plan templates (57% recording of service user/carer views and 68% co-production). The reason for this is unknown and worthy of further exploration, as amendments to the care plan template to include these two areas could potentially drive a change in clinicians’ care planning behaviour.

### 4.2. Design

The language and labelling of the care plan template along with the logo presence were the main areas for change in the evaluation. It is noted that the results were amber rather than red, suggesting that this was not a consistent issue across the care plan templates. The results highlighted several typographical errors in the care plan template. There were examples of jargon as well as acronyms being used; both can render the care plan template inaccessible and may be examples of where the principles around shared decision making are not met through access to information being denied [38]. Lin, Renwick [39] found that clinicians were not consistently familiar with shared decision making, requiring additional training. The care plan template, in this instance, can be a ‘nudge’ (without forbidding options or changing incentives [40]) to changing practice, and it behoves us to consider the most appropriate clinician in working on developing the care plan template.

The use of personal pronouns scored the lowest of the design aspects, at 55%. The use of personal pronouns in part signals how personal the care plan is; this, coupled with the clinical aspects, can contribute to the extent that the care plan is perceived as individualised and, according to Wyder, Kisely [41], can improve service user engagement.

### 4.3. Electronic Health Record

The data provided by the Trusts was either the care plan as viewed on the EHR (input care plan template) or a printout of a blank care plan template (output care plan template) of which several were filled with ‘dummy client or ‘test’ data. This raised questions about the quality of the data because of the way that EHR’s can be used. For example, a Trust may have submitted the input care plan template, but the version of that template as an output could be different. The difference could be due to the way that the Trust decided to create their care plan. Some care plans are literally ‘as seen’ on the EHR, simply printed out on paper, whereas others are printed out as a report, having been subject to data transformation.

Data transformation results in a bespoke design of the care plan template furnished with the data from the EHR inserted into specific fields of the report. Chen and Tu [42] identify three phases to report generation: data query, where the raw data are retrieved; data transformation, where the structure is converted to a ‘report layout’; and, finally, formatting, where specific fonts, size and colour are applied and made available for print as a PDF or similar. When applied to the EHR, data can be omitted or included in the report [26], resulting in a more tailored, user-friendly report (care plan), enhancing the service user experience. This means that the design heuristics can be optimised regardless of the data being input to the EHR; for example, the font and size (minimal 11 point) can be specified to enhance accessibility [26]. The volume of white space and grouping of information for easier reading and reduced cognitive load can be adjusted [43,44,45], and headings can be tailored to accessible language [43,45,46]. 

The variation in EHRs means that not all NHS Trusts will have the potential for a report to be written, as some do not include the report writing functionality or the version purchased may have limited functionality. Where a report is written, the possibility of omitting information could impact upon the quality of the data drawn upon for this evaluation, as the missing data may be located on the EHR. Information, such as the review date, care coordinator/lead professional contact details, carer details, safety and crisis plan and the person having a copy of their care plan, could have been evaluated as missing when it was in the EHR. Regardless of genuine missing data, or the data being suppressed in the EHR, this information is not available to the service user in their care plan. However, it is unknown whether this missing information from the care plan is made available to the service user elsewhere.

### 4.4. Care Plan Typology

**Figure 1:**
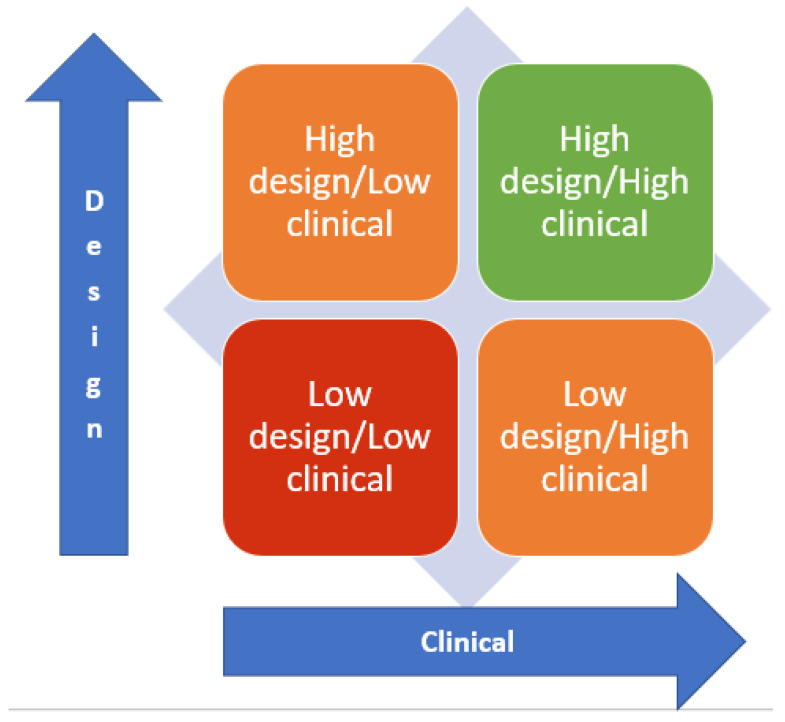
Proposed care plan typology.

A typology of the care plan template can be considered against the axis of design and clinical. Drawing upon the metrics used for the RAG rating, over 80% compliance was deemed acceptable. Thus, care plan templates can be evaluated as high (above 80%) or low (below 80%) on both the design and clinical axis—see Figure 1. Of the sample provided, the analysis found that only 11% (*n* = 5) of care plans were High Design/High Clinical, 42% (*n* = 19) were High Design/Low Clinical, 7% (*n* = 3) were Low Design/High Clinical and 40% (*n* = 18) were Low Design and Low Clinical.

NHS Trusts can apply the evaluation tool to the printed-out version of their care plan. The RAG rating will enable them to identify and focus on the areas for improvement, be it clinical, design or both aspects. The design of each Trust’s care plan template in England is likely to be unique, based upon the six different EHRs in use and the absence of a mandated care plan template. There is potential for collaborative work across Trusts using the same EHR to optimise the care plan template, improving adherence to standards and providing resource efficiency. The mandated care plan template in Wales is included in the sample and is subject to evaluation and review.

## 5. Conclusions

A typology of care plan templates is proposed based on the evaluation of 47 care plan templates in use across NHS community mental health Trusts in England and Wales. The typology defines the care plan template against the extent that it meets the established design and clinical standards. Meeting the standards at 80% or more is ‘high’ and below 80% is ‘low’. The care plan templates were collectively considered against the design and clinical standards, scoring 87% against design standards and 60% against clinical standards. Priority areas for improvement, based upon the red, amber, and green ratings, are that care plan templates include places to record the actions that service users and carers will take to contribute to their care plan and record the name and contact details for their care coordinator or lead professional and others involved in the person’s care. These areas for improvement across design and clinical aspects echo the wider research narrative on care planning. This further justifies the importance of providing clinicians with a care plan template that will optimise best practices. Following further testing of the evaluation tool, individual Trusts will be able to use the tool to evaluate their care plan templates and inform change.

This study is confined to community mental health care plans, and where the sample size against the population is large, the quality of the data provided by the NHS Trusts was variable. There is the potential to replicate the study design for care plan templates in inpatient settings.

In conclusion, there is a wide variability in the quality of care plan templates in England and Wales, and deficits are identified in design standards. The clinical standards were much worse overall than the design standard adherence. This suggests that an evidence-based typology is required to evaluate care plan templates to ensure standards of clinical practice are maintained and to enhance the practical and clinical utility of care plans in community mental health services.

Further research is needed to better understand the utility of the care plan template. The design of the template is one aspect, how it is used in practice is another, and this is, as yet, unknown.

## Figures and Tables

**Table 1:** Data collection tool for care plan clinical content.

Care Plan Clinical Content
Are the service users’ goals recorded [22]?
Does the care plan template have a place to record that the service user has been given a copy of the care plan [10,22]?
Does the care plan template have a place to record the next review date [10,22]?
Does the care plan template have a place where the service user can record their views and preferences and any differences of opinion [10,22]?
Does the care plan template invite co-production between the service user and the care coordinator/key worker [22]?
Does the care plan template invite recording of the actions that services will take, including interventions (NHSE and Improvement 2021)?
Does the care plan template invite recording of the actions that the service user (and carer if there is one) will take [10]?
Does the care plan template record the names and contact details of who is involved [22]?
Does the template include a safety and crisis plan [10,22]?
Is there a named care coordinator/key worker with contact details [10,22]?
Is there a place to identify any carers or informal sources of support (NHS England & NHS Improvement, 2021)?

**Table 2:** Care plan summary results.

Overall Results	Yes
Care Plan clinical Content	60%
Page Layout	75%
Information Layout	92%
Language and Labelling	77%
Cognitive and Memory Load	96%
Use of fonts	90%
Use of Colour	91%

Compliance percentages were then rated as amber where the results are 50% to 79% and green when the results are over 80%.

**Table 3:** Clinical summary results.

Care Plan Clinical Content	Yes
Are the service users’ goals recorded [22]?	82%
Does the care plan template have a place to record that the service user has been given a copy of the care plan [10,22]?	52%
Does the care plan template have a place to record the next review date [10,22]?	57%
Does the care plan template have a place where the service user can record their views and preferences and any differences of opinion [10,22]?	57%
Does the care plan template invite co-production between the service user and the care coordinator/key worker [22]?	68%
Does the care plan template invite recording of the actions that services will take, including interventions (NHSE and Improvement 2021)?	95%
Does the care plan template invite recording of the actions that the service user (and carer if there is one) will take [10]?	34%
Does the care plan template record the names and contact details of who is involved [22]?	45%
Does the template include a safety and crisis plan [10,22]?	64%
Is there a named care coordinator/key worker with contact details [10,22]?	57%
Is there a place to identify any carers or informal sources of support (NHS England & NHS Improvement, 2021)?	47%
Total	60%

Compliance percentages were then rated as red where the results are 0% to 49%, amber where the results are 50% to 79% and green when the results are over 80%.

**Table 4:** Page layout.

Page Layout	Yes
Does the care plan have only horizontally orientated text?	99%
Does the care plan only contain service user relevant information?	89%
Is the care plan landscape?	49%
Is the logo discrete?	51%
Is there at least a 2.5 cm white space border around the care plan?	88%
Total	75%

Compliance percentages were then rated as red where the results are 0% to 49%, amber where the results are 50% to 79% and green when the results are over 80%.

**Table 5:** Information layout.

Information Layout	Yes
Are the headings formatted the same?	90%
Are the sections of information delineated?	96%
Areas for writing are sufficient in size—allowing for large writing (14 point)?	100%
Could a care coordinator understand how to complete the care plan within 1 h?	100%
Is there a consistent pattern to the template where possible?	86%
Are words in capitals, italics and/or underlining not present?	83%
Total	92%

Compliance percentages were then green when the results are over 80%.

**Table 6:** Language and labelling.

Language and Labelling	Yes
Are the headings short, concrete and on one line?	79%
Is the care plan template free from any spelling or grammatical errors?	76%
Is the care plan template free from jargon, abbreviations, and technical language?	79%
Is the reader addressed with personal pronouns?	55%
Is the template written in plain English?	94%
Total	77%

Compliance percentages were then rated as amber where the results are 50% to 79% and green when the results are over 80%.

**Table 7:** Cognitive and memory load.

Cognitive and Memory Load	Yes
Are tick boxes used where appropriate?	96%
Total	96%

Compliance percentages were then rated as green when the results are over 80%.

**Table 8:** Use of fonts.

Use of Fonts	Yes
Are compressed fonts avoided?	100%
Are there a maximum of 2 different fonts used within the care plan template?	96%
Is the font sans serif?	95%
Is the header at least 2 points larger?	61%
Is the text at least 12 points?	96%
Total	90%

Compliance percentages were then rated as amber where the results are 50% to 79% and green when the results are over 80%.

**Table 9:** Use of colour.

Use of Colour	Yes
Is there high contrast between the background and font colour?	91%
Total	91%

Compliance percentages were then rated as green when the results are over 80%.

## Data Availability

Freedom of information data can be sought by contracting the participating NHS Trusts. Please contact the lead author as appropriate.

## Supplementary Materials

The following supporting information can be downloaded at: https://www.mdpi.com/article/10.3390/nursrep14010026/s1, Table S1. Nielson’s Ten Usability Heuristics.

## Public Involvement Statement

There was no public involvement in any aspect of this research.

## Guidelines and Standards Statement

This manuscript was drafted against the SQUIRE [28] reporting guidelines for quality improvement in health care.
