# Supplementary material for: Care Plan Templates in Adult Community Mental Health Teams in England and Wales: An Evaluation
Source: Nurs Rep. 2024 Feb 1;14(1):340–52. doi: 10.3390/nursrep14010026 (PMC10885041; doi:10.3390/nursrep14010026)
Supplement: Supplementary file 1 [file nursrep-14-00026-s001.zip › nursrep-2733921-supplementary.pdf]

Supplementary Material

Table S1. Nielson's Ten Usability Heuristics.

Heuristic	Principle	Descriptor
1	Visibility of system status	The system should always keep users informed about what is going on, through appropriate feedback within reasonable time.
2	Match between system and the real world	The system should speak the users' language, with words, phrases and concepts familiar to the user, rather than system-oriented terms, and follow real-world conventions, making information appear in a natural and logical order.
3	User control and freedom	Where the user chooses a system function in error, they should have a clearly marked 'exit' without having to run through extended dialogue. Undo and redo functions should be available to users.
4	Consistency and standards	Users should not have to wonder whether different words, situations, or actions mean the same thing. Typical platform/user interface conventions should be used.
5	Error prevention	Even better than good error messages a careful design, which prevents a problem from occurring in the first place. Either

		eliminate error-prone conditions or check for them and present users with a confirmation option before they commit to the action.
6	Recognition rather than recall	Minimize the user's memory load by making objects, actions, and options visible. The user should not have to remember information from one part of the interface to another. Instructions for use of the system should be visible or easily retrievable whenever appropriate.
7	Flexibility and efficiency of use	Accelerators, unseen by the novice user, may often speed up the interaction for the expert user such that the system can cater to both inexperienced and experienced users. Allow users to tailor frequent actions.
8	Aesthetic and minimalist design	Dialogues should not contain information which is irrelevant or rarely needed. Every extra unit of information in a dialogue competes with the relevant units of information and diminishes their relative visibility.
9	Help users recognize, diagnose, and recover from errors	Error messages should be expressed in plain language (no codes), precisely indicating the problem, and constructively suggest a solution.
10	Help and documentation	Even though it is better if the system can be used without documentation, it may be

	necessary to provide help and documentation. Any such information should be easy to search, focused on the user's task, list concrete steps to be carried out, and not be too large.
